# Early childhood educators’ perceptions of preschoolers' mental health problems: a qualitative analysis

**DOI:** 10.1186/1744-859X-13-1

**Published:** 2014-01-03

**Authors:** George Giannakopoulos, Eirini Agapidaki, Christine Dimitrakaki, Despoina Oikonomidou, Dimitra Petanidou, Lia Tsermidou, Gerasimos Kolaitis, Yannis Tountas, Kalliroi Papadopoulou

**Affiliations:** 1 Department of Hygiene, Epidemiology and Medical Statistics, Center for Health Services Research, Athens University Medical School, Athens 11527, Greece; 2 Faculty of Early Childhood Education, Athens University School of Education, Athens 10680, Greece; 3 Department of Child Psychiatry, Athens University Medical School, Aghia Sophia Children's Hospital, Athens 11527, Greece

**Keywords:** Early childhood education, Mental health, Preschoolers

## Abstract

**Background:**

Early childhood education services create potentially optimal opportunities to identify and respond effectively to preschoolers' mental health problems. However, little is known about the knowledge, skills and competencies of early childhood educators in the area of mental health. The present study aimed to contribute to this field through conducting focus group interviews with professionals from public early childhood education centres in Greece.

**Methods:**

Thirty-four educators attended five focus group meetings, with each group consisting of five to nine participants and two discussion facilitators. A thematic analysis was conducted using line-by-line open coding. Constructed codes from the wording used by the participants in the interviews were created, and constant comparisons for developing themes as well as seeking data not conforming to each theme were used independently by two researchers. At the end of this process, no new information was being provided and there was repetition in each of the categories.

**Results:**

The analysis identified three themes in the data: risk factors for preschoolers' mental health problems, signs of preschoolers' mental health problems and practices of helping preschoolers with mental health problems. Results suggested that early childhood educators had satisfactory awareness of many preschoolers' mental health issues, although they showed a rather limited understanding in some domains. Moreover, they seemed to deliver inadequate practices in responding effectively to children's and families' mental health problems.

**Conclusions:**

Best practice training in working with preschoolers, families and mental health services seems essential for helping young children receive the best level of support through early identification and intervention services for possible mental health problems.

## Background

During the preschool years (ages 2 to 5 years old), brain development shows some of its most active and sophisticated advances, and many mental competences are expressed for the first time [1]. Behaviours related to temperament, attachment, frustration, empathy and aggression are elaborated in this early stage in the context of the interplay between genes and environment [2].

Although there is relatively limited evidence on preschool psychopathology compared with epidemiologic studies of psychiatric disorders in older children, the current research suggests with certainty that the rates of the common psychiatric disorders in preschoolers are similar to those seen in later childhood [3]. In early childhood, mental health problems primarily consist of behaviour and emotional problems. These affect approximately one in every seven children. Oppositional defiant disorder, attention deficit/hyperactivity disorder (ADHD), depression, separation anxiety disorder and parent–child relationship problems are the most commonly diagnosed mental health problems in preschool populations.

Left untreated, up to 50% of preschool problems continue through the childhood years [4]. Because of their high prevalence and persistence into later life, community-based approaches are needed to reduce their associated burden in a range of areas (e.g. psychosocial functioning and cognitive performance). In fact, there is credible evidence that intervening during preschool years can have a greater cost-effective impact on multiple developmental outcomes, compared to later school or adult intervention [5-8]. Efficacious promotive and preventive interventions have been developed mainly for behaviour and, to a lesser extent, emotional problems and could be diffused from research to everyday practice, assuring fidelity to original intervention implementation [9].

Early childhood education services create potentially optimal opportunities to identify and respond effectively to early mental health problems in a feasible way, given the large number of children who attend. However, little is known about the knowledge, skills and competencies of early childhood educators in the area of child mental health. It has been shown that only a minority of preschoolers with mental health problems are referred by child care services for further assessment and treatment [10]. An Australian study tried to explore childcare educators' and managers' understanding of child and parental mental health and the early signs of mental health problems [11]. Qualitative analysis of semi-structured interviews showed that childcare staff were able to explain child wellbeing but were somewhat limited in their knowledge of risk and protective factors for child and parental mental health. The educators seldom mentioned the impact of broader community and societal issues on mental health, and they tended to attribute mental health problems to family violence or poor parenting. Moreover, they did not link mental health promotion to the principles of the early years education curriculum. Nevertheless, many of them indicated a desire for further training in mental health.

Also, another study in Australia aimed to identify the strategies, facilitators and key challenges for promoting children's social and emotional wellbeing as reported by childcare directors and workers during semi-structured interviews [12]. They reported mainly informal individual caring and helping strategies with few centre-wide policies or systematic scheme-wide strategies. Frequent difficulties communicating with non-English speaking parents and/or children, lack of staff training and inadequate resources for activities were the key challenges identified by the participants. Perceived facilitators included staff having strong relationships with each other and sharing a common philosophy, as well as having an open door policy for parents. A subsequent qualitative analysis of semi-structured interviews by the same researchers revealed that family day care educators were not enough comfortable identifying causes and early signs of mental health problems [13]. Limited financial resources, lack of training and hesitance raising child mental health issues with parents were the most common barriers that were reported by the participants.

The present study aimed to replicate the findings from the abovementioned qualitative studies and to contribute to the scarce literature in this field and inform education and health policies through exploring early childhood educators' perceptions of risk factors and early signs of preschoolers' mental health problems and practices of responding to these difficulties. For this reason, focus group interviews with professionals from Greek public early childhood education centres were conducted.

## Methods

### Participants and procedure

The present study was part of the project entitled ‘Promotion of Child Psychosocial Development and Prevention of Difficulties in the Childcare Context’ under the Action ‘Excellence’ of the operational program ‘Education and Lifelong Learning’. The provision of Greek public early childhood education is offered by municipalities at no cost to low-income families or at a low monthly cost to medium- and high-income families. However, public services cannot respond to all applications, and a proportion of children attend private education centres with state funding. In the present study, early childhood educators were recruited from six municipalities in Athens, Greece, that included socially disadvantaged areas and were proximal to the venue of the focus group meetings. Within each municipality, the person being in charge of the public preschool education centres was contacted via telephone calls in order to forward to the childcare centres written invitations for the group discussions. Through the invitations, educators were asked to contact the research team members so as to express their interest for participation. To be eligible for the study, preschool educators had to have at least 1 year of experience and hold at least a 2-year vocational degree. It was ensured that no more than two staff members from each centre would be included. After recruitment, 48 educators agreed to take part in the study, and 34 of them finally attended the focus group meetings. All participants were female. Six participants held a 2-year vocational training in preschool education, 16 participants held a college degree (4-year training) in preschool education, 11 participants held a university degree (4-year training) in early childhood education and 1 participant held a masters degree in early childhood education. In general, this distribution of educational level reflects the situation in the entire population of early childhood educators in Greece, where vocational training and higher education degree are required for appointment as an assistant educator or an educator, respectively. In the present sample, 3 educators had less than 2-year experience, 9 educators had 2- to 10-year experience, 15 educators had 10- to 20-year experience, and 7 educators had more than 20-year experience.

Five focus group meetings were conducted within a 2-week period during June 2013. Each group consisted of five to nine educators and two discussion facilitators. The average duration of each focus group discussion was approximately 120 min. A focus group discussion guide was developed, pilot tested and reviewed before the final administration. The participants were given a brief definition of child mental health as follows: ‘The achievement of expected developmental cognitive, social, and emotional milestones and by secure attachments, satisfying social relationships, and effective coping skills’. They were then asked an open set of questions related to the aims of the present research: (1) *Tell me what you think are the causes of mental health problems in children?*, (2) *Tell me what you think the early signs of a mental health problem are for children? (Prompt: what might they behave like with other children?)* and (3) *If you suspect a child has a mental health problem, what do you do?* The participants also completed a demographic questionnaire. Focus group discussions were audio-recorded and transcribed verbatim. Ethics approval was granted from the Bioethics Committee of the University of Athens Medical School.

### Data analysis

A thematic analysis was conducted in order to explore the educators' understanding and practices regarding preschoolers' mental health problems using line-by-line open coding [14,15]. Concepts emerged from the raw data and later were grouped into conceptual categories. Constructed codes from *in vivo* codes (the wording used by participants in the interviews) were created, based mainly on a comprehensive model of understanding child mental health needs [16]. Constant comparisons for developing themes and seeking data not conforming to each theme were used independently by two researchers. At the end of this process, no new information was being provided and there was repetition in each of the categories.

## Results

The analysis identified three themes in the data: risk factors for preschoolers' mental health problems, signs of preschoolers' mental health problems and practices of helping preschoolers with mental health problems. Each theme is reported below, and its content is illustrated with quotes from the participants.

### Risk factors for preschoolers' mental health problems

Inconsistent care giving was the most frequently reported risk factor for preschoolers' mental health problems. Family history of mental health problems; lack of warm, trusting and supportive relationships with significant adults; unstable home environment (e.g. family violence and parental conflict) and exposure to major stressful life events were also referred by many educators.

Hereditary factors, lack of love, affection and emotional nurture, a disturbed family environment, overprotection and oppression of children, low self-esteem and self-confidence due to impaired parent–child attachment. (Participant 5)

Each case is unique … in general, when there is tension between parents at home … when they cannot communicate effectively … they argue a lot, feel unbearable anxiety in everyday life with their kids, parents who are not stable, they do not set limits to kids. I believe parent behavior is by far the most important factor for child development in order for the child to grow up emotionally and not develop such problems. (Participant 12)

Maybe some events. For example, bereavement… such things. A divorce - something more common. (Participant 23)

A group of participants identified parent mental health problems in conjunction with lower levels of parental education, income and employment as important risks for children's mental health problems.

I believe that nowadays parents’ anxiety about the economic situation is the most important factor. I would say that all this stress that parents suffer… children are aware of this stress and the parents cannot understand that their kids from a very young age are able to understand anything that happens at home… Maybe parents talk to each other in front of their kids and children internalize this anxiety… unexpectedly. (Participant 15)

Only one educator spoke of preschoolers with limited experiences of social interaction who were at risk for mental health problems, and another participant referred to physical health problems as a risk factor for mental health problems. Finally, the only individual risk factor that was discussed was the child's impaired brain development.

### Signs of preschoolers' mental health problems

Educators felt that a preschooler who was often challenging, refusing to obey parents or carers, did not play with other children and did not enjoy being with children might face a mental health problem.

Their inability to follow simple instructions… for example, it is time for drawing, for play, it is time for having lunch or washing our hands… very simple instructions that they cannot follow, they cannot follow the rest… they cannot concentrate on something and sit at the table for long. (Participant 6)

This is a melancholic child… isolated… who does not play with other children or even on his own… To me, this child sits in a corner, all alone, without toys and children around them… or even if there are toys or kids, this child seems indifferent… to reach them or to be reached. (Participant 30)

A group of participants described preschoolers not being able to be understood by someone they did not know, not asking questions, having ongoing fears that prevented enjoyment of life and showing unprovoked anger or violence towards other children.

A child who does not respect their peers or the group… they constantly hit other kids and they become the centre of attention… a big problem… An overactive, aggressive child… (Participant 18)

Other views were that preschoolers who revert to soiling or wetting after being toilet-trained, cannot separate from parents or carers even with support and have difficulty in understanding and responding to another person's point of view or feelings may face mental health difficulties. Only one educator identified significant sleeping problems, waking a lot at night and not seeking parents or carers for comfort as signs of preschoolers' mental health problems. Additionally, just one educator described children with mental health problems as possibly very dependent on a dummy or comfort object after 4 years.

### Practices of helping preschoolers with mental health problems

The participants felt that their current practice in helping preschoolers with mental health problems included mainly taking time to observe preschoolers' behaviours and social relationships at the service and discussing these with parents.

We observe the child over a long period and during the programme of the centre… We discuss with parents about their child’s behavior and try to find together management strategies. (Participant 1)

This is a very sensitive subject. You should be clear towards the parents regarding your concerns, still in a discreet manner… without insulting them. It is helpful to suggest that they see a mental health professional in order to deal more appropriately with the problem. (Participant 19)

Developing relationships with local mental health services to support their work with preschoolers and families and having regular meetings and follow-ups with parents to support a child who may be experiencing mental health problems were the other two practices referred by the educators.

First, we ask for professional advice. There is a psychologist working for the municipal consultation service. Also, we encourage parents to seek professional help. We both get help this way… However, it depends on parents, how much ready they are to accept what you tell them. Sometimes parents do not accept our concerns at all! (Participant 30)

## Discussion

The present qualitative content analysis showed that several educators did express their knowledge about many the most important parenting and family risk factors for preschoolers' mental health problems [17]. However, their understanding about these proximal risks seemed limited in that they tended to feel that parents were to blame for their offsprings' mental health problems. This understanding may substantially impair the relationships between preschool education centres and families, increase stress and feelings of guilt in parents and ultimately may adversely affect parenting ability and children's outcomes [18]. In addition, other significant parameters such as limited family social support networks, child's difficult temperament and early separation from primary caregiver [3,19] were not mentioned by any participant.

Moreover, several educators did seem knowledgeable of key signs of common mental health problems in preschoolers. More specifically, many participants managed to identify signs of externalizing problems (e.g. oppositional defiant behaviour, inattention and hyperactivity). However, educators referred, to a lesser extent, to signs of internalizing problems (e.g. depression and separation anxiety), relationship problems (e.g. disorganized attachment and autistic features) and regulatory problems (e.g. sleep disturbances, poor self-soothing and disordered eating patterns). This fact is rather problematic, given that emotional and behavioural problems if left undetected and untreated during the preschool years create a developmental pathway that leads to internalizing and externalizing problems into the adolescent years and early adulthood [20,21].

With regard to current practices in helping preschoolers with mental health problems, the analysis showed that on an individual level, many educators take time to observe children's behaviours at the service and discuss these with parents and, to a lesser extent, work in partnership with families and local mental health services. However, it is clear that no policies and procedures (e.g. help-seeking policies, inclusive referral pathways, hosting events with professional services and coordinated interventions) are currently being developed within the early childhood education centres for responding to children with mental health problems. Moreover, the educators seem not to realize their important role in helping children with mental health problems learn new skills that support their development.

The focus group interview method provided an effective opportunity to collect rich and comprehensive material. However, despite the wealth of material, most of the statements relied on the participants' subjective perceptions. Further research in this area should therefore combine interviews with observations in everyday early childhood education practice in order to examine more reliably educators' understanding of specific real-world cases of children displaying or not displaying difficulties and the actual strategies that are employed. Additionally, the aim of the present study was the provision of critical information rather than the generalizability of the results. A purposive and of maximum variation sampling was used to ensure ‘information-rich cases’. This information may be helpful for researchers, and decision makers, in order to develop mental health promotion interventions within the early childhood care context. It cannot be claimed that the opinions reported by preschool educators are representative of the entire respective population. The study was conducted in the context of an urban area, and professionals from rural areas could have expressed different views—possibly due to factors such as less workload, lack of mental health services and fewer opportunities for continuing education. Moreover, the focus group discussions were conducted out of the educators' working hours, in a distant setting from their workplace, which means that the participants were probably those who were more aware of the importance of the issues under study. The provision of incentives for participation, the more active recruitment process and the inclusion of rural areas may achieve a better representation of the respective population in future research.

Despite its limitations, the present study revealed that early childhood educators have a limited understanding about preschoolers' mental health issues and deliver inadequate practices in responding effectively to children's and families' mental health problems. Best practice training for early childhood educators in working with preschoolers, families and mental health services seems essential for helping young children receive the best level of support during their early development. Best practice training could include (1) promoting feelings of belonging and connection for all children, families and staff; (2) developing children's social and emotional skills, such as self-regulation, relating to others, resolving conflict and feeling positive about themselves and the world around them; (3) sharing important information about the lives of the children and linking parents and carers with appropriate information and education about parenting, child development and children's mental health; and (4) helping children who are experiencing mental health problems. The latter could be enhanced though training focused on understanding mental health, promoting children's mental health, managing feeling and behaviours, managing trauma and ways to recover, seeking help and accessing support [22]. Relevant training can equip early childhood education with knowledge, attitudes and skills that are needed to identify children experiencing mental health problems, discuss effectively with parents about mental health and help seeking, and provide invaluable opportunities for early intervention.

## Competing interests

The authors declare that they have no competing interests.

## Authors’ contributions

GG participated in the design of the study, made the analyses and prepared the manuscript. EA participated in the design of the study and assisted in the analyses and manuscript preparation. CD, YT and KP designed the study and consulted on the analyses. DO, DP and LT participated in the design and the implementation of the study. GK consulted on the analyses. All authors read and approved the final manuscript.
